# Supplementary material for: The association between Alu hypomethylation and the severity of hypertension
Source: PLoS One. 2022 Jul 8;17(7):e0270004. doi: 10.1371/journal.pone.0270004 (PMC9269909; doi:10.1371/journal.pone.0270004)
Supplement: S1 Fig — (PDF) [file pone.0270004.s001.pdf]

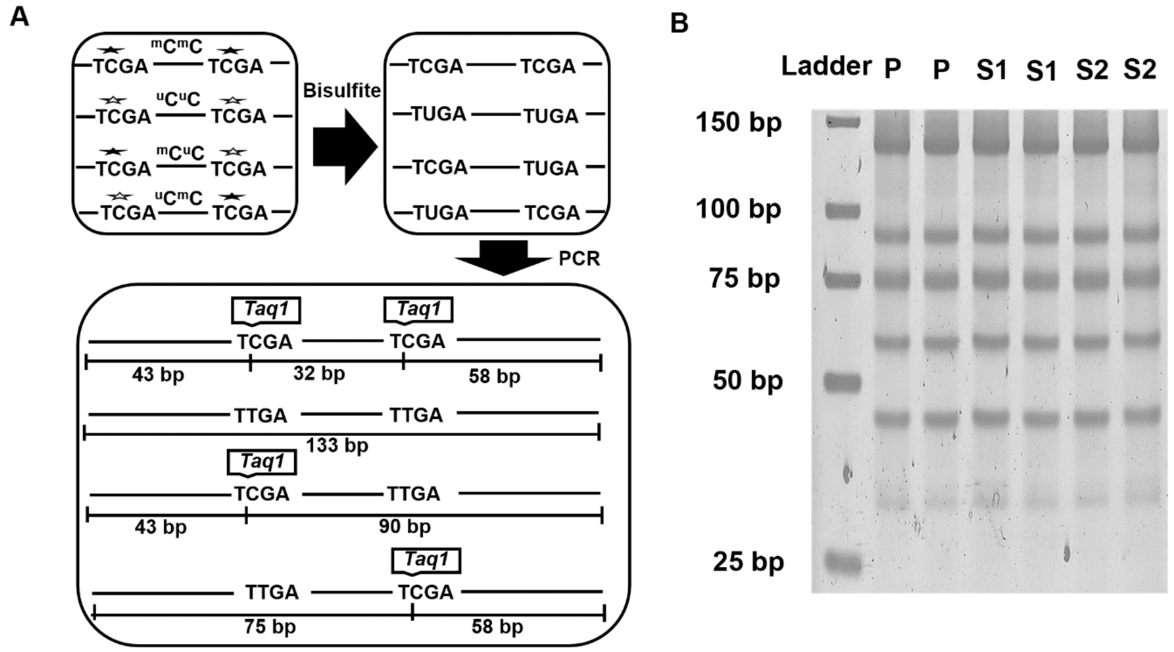

S1 Fig. COBRA-Alu assay and the methylation patterns of Alu amplicons. (A) Alu methylation patterns of COBRA-Alu assay, including fully methylated loci ($^{m}C^{m}C$), unmethylated loci (^{u} C^{u} C), and two partially methylated forms ($^{m}C^{u}$ C and $^{u}C^{m}C$). The Alu amplicons are 133 bp and contain 2 CpG-dinucleotides. After bisulfate treatment, methylated cytosine bases are not changed to uracil bases, whereas the unmethylated cytosine bases are converted to uracil bases. The PCR products are digested with *Taq1* restriction enzyme, Alu amplicons were 133, 90, 75, 58 and 43 bp. (B) Representative gel image for COBRA-Alu assay. Lane 1: Low Molecular Weight DNA Ladder (Ladder), Lane 2-3: Positive control (P), Lanes 4-5: DNA from sample 1 (S1), Lane 6-7: DNA from sample 2 (S2).
