# Supplementary material for: The association between Alu hypomethylation and the severity of hypertension
Source: PLoS One. 2022 Jul 8;17(7):e0270004. doi: 10.1371/journal.pone.0270004 (PMC9269909; doi:10.1371/journal.pone.0270004)
Supplement: S2 Fig — (PDF) [file pone.0270004.s002.pdf]

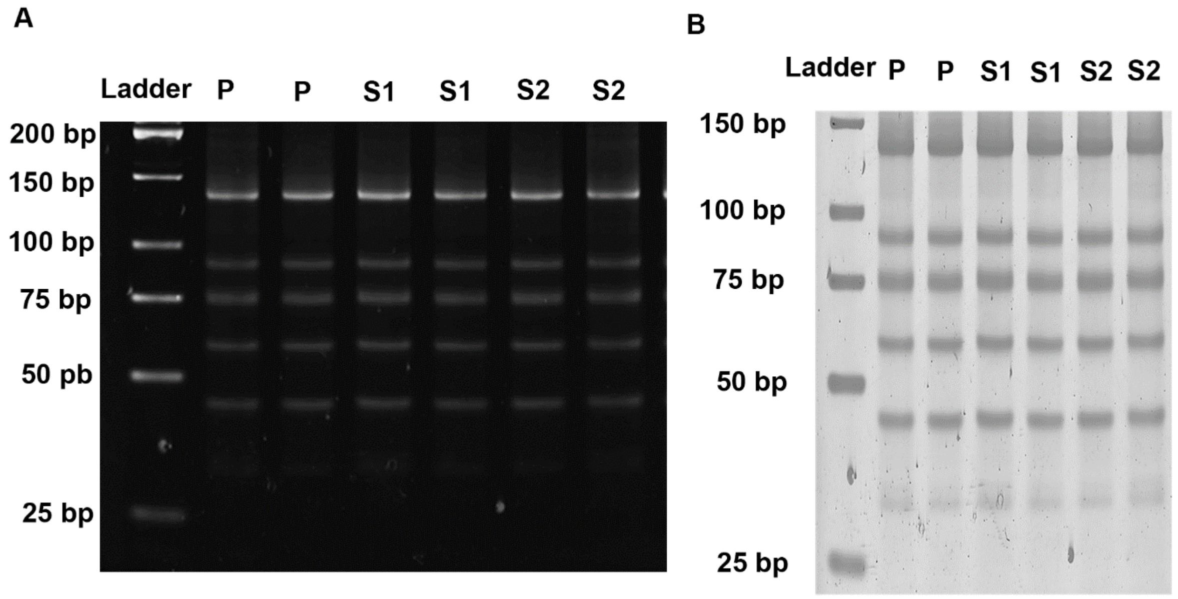

S2 Fig. The original blot and gel image. (A) The original blot of Alu amplicons were 133, 90, 75, 58 and 43 bp. (B) The gel image, Lane 1: Low Molecular Weight DNA Ladder (Ladder), Lane 2-3: Positive control (P), Lanes 4-5: DNA from sample 1 (S1), Lane 6-7: DNA from sample 2 (S2).
