# Supplementary material for: The association between Alu hypomethylation and the severity of hypertension
Source: PLoS One. 2022 Jul 8;17(7):e0270004. doi: 10.1371/journal.pone.0270004 (PMC9269909; doi:10.1371/journal.pone.0270004)
Supplement: Attachment — Submitted filename: Response to Reviewers.doc [file pone.0270004.s003.doc]

Dear Editor,

We appreciate the reviewers’ comments and have revised our manuscript accordingly. We list our responses in a point-by-point fashion and all the changes in the manuscript text are highlighted in yellow.

Reviewer #1: Overall a nice study on a clinically relevant topic. The manuscript requires further clarification on certain areas (see below). Overall it is a well written paper.

**Reply:** Thank you very much.

Ln41-42; differentiate between genetically inherited risk and epigenetic alterations over time (is there a trans-generational risk conferred of epigenetic alterations?). Sentence is ambiguous.

**Reply:** Thank you very much. Here in this version, we have edited the word form “inherited genetic component” to “epigenetic alterations” (page 2 line 42)

Denote and define abbreviations before use in text of manuscript.

**Reply:** We would like to thank reviewer 1 for suggesting additionally defined abbreviations in our manuscript. In this version, we added more defined abbreviations in this manuscript: diabetes mellitus (DM), Combined Bisulfite Restriction Analysis (COBRA), Cytosine-phosphate-guanine (CpG), hemoglobin A1c (HbA1C), and small interfering RNA (siRNA). (page 2 line 60, page 3 line 77, page 4 line 109-110, page 9 line 259 and 265, respectively)

How was blood pressure measured- was it 24hour ambulatory blood pressure, or static (if so when was it measured and was this kept constant).

**Reply:** Thank you very much. The participants in this study were chosen by the family care team, and blood pressure was monitored weekly to keep constant levels. Furthermore, we also took the blood pressure of the sample for an hour before beginning our experiment.

Materials and Methods

This section requires editing and improvement. The reader must be well informed of the methods used and how they were undertaken

e.g. How were the white blood cells isolated or were they (whole blood used?). Were leucocytes immuno-isolated by magnetic separation? Better description of the BP analysis needed and classification of cohorts. Were CBCs and blood indices recorded? Better description of Alu methylation calculations required also reference needed for the original paper(s) describing the technique.

**Reply:** Thank you very much for this valuable advice. We updated the Materials and Methods to explain that DNA was extracted from the buffy coat by centrifuging whole blood, using lysis buffer and proteinase K digestion, and phenol-chloroform extraction protocols. Please see the DNA extraction and Bisulfite DNA modification topic in the Materials and Methods (page 4 line 97-101). Furthermore, the reference for the description of Alu methylation calculations has already been added. (page 5 line 142)

Ln114-116; syntax requires revising

**Reply:** We apologize for this mistake. The sentence has been edited from "COBRA results were categorized into four groups based on the status of the methylation at the two CpG dinucleotides” to “Methylation patterns were classified into four groups based on the COBRA results of the two CpG sites. (page 5 line 129-131)

Ln152-153; error

**Reply:** We apologize for this mistake. The word has been edited from “a significantly an inverse” to “significantly inverse” (page 6 line 168)

Ln178; error

**Reply:** We apologize for this mistake. The word has been edited from “hypertensiove” to “hypertensive” (page 7 line 196)

Ln179; error

**Reply:** We apologize for this mistake. The word has been edited from “were” to “was” (page 7 line 197)

Reviewer #2: The manuscript by Thongsroy and Mutirangura investigates the interesting possibility of an association between hypertension and hypomethylation of Alu repeats. The authors are not sufficiently careful in their scientific statements and their methodology is unconvincing.

Abstract. Alu methylation cannot prevent DNA damage unlike what is stated. There are very many types of DNA methylation which have no relationship to Alu repeats. In addition, the authors need to distinguish in the Abstract between unrepaired or persisting DNA damage and DNA not being accessible to certain types of DNA damage. It is essential that age be mentioned in the Abstract.

**Reply:** Thank you very much, Alu methylation preventing DNA damage was reported as one of the mechanisms how DNA methylation prevents genomic instability (Patchsung, Settayanon et al. 2018). The underlying mechanism of this phenomenon is the role of natural occurring hypermethylated DNA gaps in relieving DNA double helix torsional force (Yasom, Watcharanurak et al. , Pornthanakasem, Kongruttanachok et al. 2008, Thongsroy, Patchsung et al. 2018). This mechanism was demonstrated to prevent all kinds of DNA damage (Yasom, Watcharanurak et al.). Therefore, In this version, we added this paragraph in the introduction (page 2 line 50-54)

References

1. Patchsung, M., et al., Alu siRNA to increase Alu element methylation and prevent DNA

damage. Epigenomics, 2018. 10(2): p. 175-185.

2. Pornthanakasem, W., et al., LINE-1 methylation status of endogenous DNA double-strand

breaks. Nucleic acids research, 2008. 36(11): p. 3667-3675.

3. Thongsroy, J., et al., Reduction in replication‐independent endogenous DNA double‐strand

breaks promotes genomic instability during chronological aging in yeast. The FASEB

Journal, 2018. 32(11): p. 6252-6260.

4. Yasom, S., et al., The roles of HMGB1‐produced DNA gaps in DNA protection and aging

biomarker reversal. FASEB BioAdvances.

Lines 50 -52. Association does not indicate causality. “Therefore” should be deleted.

**Reply:** Thank you very much. Here in this version, we added the mechanisms supporting the Alu methylation preventing DNA damage in the introduction. (page 2 line 50-54)

Line 56. “Significant” should be used only for a statistical test or modified by “biologically.” Do they mean “There was signifant hypomethylation in DM patients compared to ….”

**Reply:** Thank you very much. The sentence was edited from “there were significant Alu hypomethylation levels in type 2 DM” to “there were significantly decreased Alu hypomethylation levels in type 2 DM **when compare with normal controls**” (page 3 line 62)

Line 66. Whenever the authors refer to epigenetics or transcription, the type of tissue or cell must be specied and for DNA methylation the type of analysis (overall levels, methylation at specific gene promoters, etc.).

**Reply:** Thank you very much. Here in this version, we have added the type of cell and type of analysis in the sentence “Recent results found that diastolic pressure was associated with DNA methylation **of white blood cell**, whereas systolic pressure was not associated with DNA methylation **when using Epigenome-wide association study analysis.** (page 3 line 71-72)

Line 137. Figures 1, 2, 4. All data should be age adjusted, not just some of it. All comparisons (not just some) should be between individuals of the same age group unless the effect of age is being determined. It is well established that DNA methylation levels change with age. The authors should have separately analyzed the age effect among their normal controls.

**Reply:** Thank you very much. Fig 3 was the graph after the adjusted age of Fig 1, and Figs 4C and 4D were the graphs after the adjusted age of Figs 4A and 4B, respectively. Figure 2 shows the correlation graph. In this version, we included age information in Figure 2 by adding the colors to distinguish different age groups

Line 246. The authors should be much more careful in their scientific statements. What is “improve Alu methylation”? How could medicine target Alu methylation in blood vessels? There are so many sources of changes in blood pressure that do not involve DNA damage that the authors are ignoring.

**Reply:** Thank you very much. In this version, we added previous evidence supporting Alu hypomethylation might promote accumulated DNA damage leading to genomic instability by loss of cellular aging function in patients with hypertension in the discussion. (page 9 line 264-272)

“Our previous publication demonstrated that Alu hypomethylation was associated with increasing endogenous DNA damage. Additionally, Alu small interfering RNA (siRNA) could increase Alu methylation, decrease endogenous DNA damage, and increase cell viability (Patchsung, Settayanon et al. 2018). In this study, we observed Alu hypomethylation in hypertensive patients. So, Alu hypomethylation accumulating DNA damage may lead to cellular aging vessels of hypertensive patients. Here, we hypothesize that Alu siRNA might increase Alu methylation, resulting in decreased genomic instability and reduced blood pressure status in hypertensive patients. Therefore, Alu methylation-editing technology, such as Alu siRNA, may play a role in the treatment of hypertensive patients.”

Reference

1. Patchsung, M., et al., Alu siRNA to increase Alu element methylation and prevent DNA

damage. Epigenomics, 2018. 10(2): p. 175-185.

evidence do they have that TaqI digestion was always complete? This is important because of the small differences they are interpreting.

They need technical duplicates or triplicates to show no differences.

They should display a stained gel so that the reader can see the resolution obtained for these small bands.

**Reply:** Thank you very much. The below figure shows the duplicate samples that showed no differences. The same patterns of the samples were digested by TaqI digestion. Furthermore, we used the same positive control to adjust inter-assay variation. (page 5 line 142). In this version, we have already added this stained gel figure in Supplement Figure 1B (S1 fig).

Lane 1: Low Molecular Weight DNA Ladder (Ladder), Lane 2-3: Positive control (P), Lanes 4-5: DNA from sample 1 (S1), Lane 6-7: DNA from sample 2 (S2).
