# Supplementary material for: The association between Alu hypomethylation and the severity of hypertension
Source: PLoS One. 2022 Jul 8;17(7):e0270004. doi: 10.1371/journal.pone.0270004 (PMC9269909; doi:10.1371/journal.pone.0270004)
Supplement: Attachment — Submitted filename: Response to Reviewers.doc [file pone.0270004.s004.doc]

Dear Editor,

We appreciate the reviewers’ comments and have revised our manuscript accordingly. We list our responses in a point-by-point fashion and all the changes in the manuscript text are highlighted in yellow.

Reviewer #1: The authors have addressed my comments. Reviewer 2 raised some very valuable and pertinent comments, but I see the authors have addressed these, backed by referencing (Alu methylation prevents DNA damage and how DNA methylation prevents genomic instability).

**Reply:** Thank you very much.

Reviewer #3: This paper by Thongsroy and Mutirangura is a revision of a paper that I did not have as a first reader. It attemps to relate the Alu methylation status with hypertension severity using the COBRA approach. It seems that Alu methylation is inversely proportional to diastolic or systolic blood pressure.
I have no major observation concerning the scientific message. The authors took into account the influence of sex of diabetes mellitus. They also analyze the follow-up of patients on a 5 years period (2015-2019), which is one of the most original part of the study. It seems that the age decreases the Alu methylation but not in the pre-hypertensive group, which would warrant an interpretation.
In sum, I would recommend a careful editing of the English by a native English speaker or a professional service. With an adequate discussion about this question of Alu methylation not changed during ageing in the pre-hypertensive group, I’ll recommend publication of this paper.

**Reply:** Thank you very much. Here in this version, we have added the sentence “There was no significant difference in prehypertension. Thus, the number of follow-up samples with prehypertension was lower than in the other groups. These limit the use of Alu methylation levels directly as a biomarker for clinical application.” in the discussion part (page 8 lines 230-232). This manuscript was edited by one or more of the the highly qualified native English speaking editors at American Journal Experts (AJE).
